# Impact of isoniazid preventive therapy on the evaluation of long-term effectiveness of infant MVA85A vaccination

**DOI:** 10.5588/ijtld.16.0709

**Published:** 2017-07

**Authors:** E. W. Bunyasi, A. K. K. Luabeya, M. Tameris, H. Geldenhuys, H. Mulenga, B. S. Landry, T. J. Scriba, B-M. Schmidt, W. A. Hanekom, H. Mahomed, H. McShane, M. Hatherill

**Affiliations:** * South African Tuberculosis Vaccine Initiative, Institute of Infectious Disease and Molecular Medicine, and Division of Immunology, Department of Pathology, University of Cape Town, Cape Town, South Africa; † Aeras, Rockville, Maryland, USA; ‡ Department of Social and Behavioral Sciences, School of Public Health and Family Medicine, University of Cape Town, Cape Town; § Department of Health, Western Cape and Division of Community Health, Stellenbosch University, Stellenbosch, South Africa; ¶ Jenner Institute, Nuffield Department of Clinical Medicine, University of Oxford, Oxford, UK

**Keywords:** children, immunisation, treatment, IPT, South Africa

## Abstract

**SETTING:**

South Africa.

**OBJECTIVE:**

To evaluate the long-term effectiveness of infant modified vaccinia Ankara virus-expressing antigen 85A (MVA85A) vaccination against tuberculosis (TB).

**DESIGN:**

We analysed data from a double-blind randomised placebo-controlled Phase 2b MVA85A infant TB vaccine trial (2009–2012), with extended post-trial follow-up (2012–2014). Isoniazid preventive therapy (IPT) was provided by public health services according to national guidelines. The primary outcome was curative treatment for TB disease. Survival analysis and Poisson regression were used for study analysis.

**RESULTS:**

Total follow-up was 10 351 person-years of observation (pyo). Median follow-up age was 4.8 years (interquartile range 4.4–5.2). There were 328 (12%) TB cases. TB disease incidence was 3.2/100 pyo (95%CI 2.8–3.5) overall, and respectively 3.3 (95%CI 2.9–3.9) and 3.0 (95%CI 2.6–3.5)/100 pyo in the MVA85A vaccine and placebo arms. A total of 304 children (11%) received IPT, with respectively 880 and 9471 pyo among IPT and non-IPT recipients. There were 23 (7.6%) TB cases among 304 IPT recipients vs. 305 (12.9%) among 2374 non-IPT recipients (*P* = 0.008). IPT effectiveness was 85% (95%CI 76–91).

**CONCLUSION:**

Extended follow-up confirms no long-term effectiveness of infant MVA85A vaccination, but a six-fold reduction in TB risk can be attributed to IPT. National TB programmes in high TB burden countries should ensure optimal implementation of IPT for eligible children.

## Abstract (FR)

**OBJECTIF:**

Evaluer l'efficacitée à long terme de la vaccination des nourrissons par le MVA85A (modified vaccinia Ankara virus-expressing antigen 85A vaccine) contre la tuberculose (TB).

**SCHÉMA:**

Nous avons analysé les données d'un essai vaccinal de phase 2b en double aveugle, randomisé, contre placebo du vaccin MVA85A contre la TB du nourrisson (2009–2012) avec un suivi prolongé après l'essai (2012–2014). Un traitement préventif par isoniazide (IPT) a été fourni par les services de santé publique en accord avec les directives nationales. Le résultat principal a été le traitement curatif de la TB maladie. Notre analyse a utilisé l'analyse de survie et la régression de Poisson.

**RÉSULTATS:**

La durée totale du suivi a été de 10 351 personnes-années d'observation (pyo). L'âge médian du suivi a été de 4,8 ans (intervalle interquartile 4,4–5,2). Sont survenus 328 (12%) cas de TB. L'incidence d'ensemble de la TB maladie a été de 3,2/100 pyo (IC95% 2,8–3,5) ; et 3,3 (IC95% 2,9–3,9) contre 3,0 (IC95% 2,6–3,5)/100 pyo, dans les bras vaccin MVA85A et placebo, respectivement. Ont reçu l'IPT 304 (11%) enfants, avec 880 et 9471 pyo parmi les enfants IPT et non IPT, respectivement ; 23 (7,6%) cas de TB sont survenus parmi 304 enfants qui ont reçu l'IPT contre 305 (12,9%) parmi les 2374 enfants qui n'ont pas reçu l'IPT (*P*=0,008). L'efficacité de l'IPTa été de 85% (IC95% 76–91).

**CONCLUSION:**

Un suivi prolongé confirme l'absence d'efficacité à long terme de l'administration du vaccin MVA85A chez les nourrissons mais une division par six du risque de TB est attribuée à l'IPT. Les programmes nationaux TB dans les pays durement frappés par la TB devraient s'assurer de la mise en œuvre optimale de l'IPT pour les enfants éligibles.

## Abstract (SP)

**OBJETIVO:**

Evaluar la eficacia a largo plazo de la vacunación antituberculosa de los lactantes con MVA85A.

**MÉTODO:**

Se analizaron los datos de un ensayo clínico aleatorizado, con doble anonimato y controlado con placebo en fase 2b de la vacuna antituberculosa MVA85A en lactantes (del 2009 al 2012), con un seguimiento prolongado después del ensayo (del 2012 al 2014). Los servicios de atención de salud del sector público suministraron el tratamiento preventivo con isoniazida (TPI) según las directrices nacionales. El principal criterio de valoración fue el tratamiento curativo de la tuberculosis (TB) activa. En el análisis estadístico se aplicaron el análisis de supervivencia y la regresión de Poisson.

**RESULADOS:**

El seguimiento total fue de 107 351 años-persona de observación (apo). La mediana de la edad del seguimiento fue 4,8 años (amplitud intercuartil [AIC] de 4,4 a 5,2). Se presentaron 328 casos de TB (12%). La incidencia global de enfermedad tuberculosa fue 3,2 por 100 apo (IC del 95% de 2,8 a 3,5); en el grupo que recibió la vacuna MVA85A la incidencia fue 3,3 por 100 apo (IC95% de 2,9 a 3,9), comparada con 3,0 por 100 apo en el grupo que recibió placebo (IC95% de 2,6 a 3,5). Trescientos cuatro niños recibieron el TPI (11%) y su seguimiento fue de 880 apo; el seguimiento de quienes no recibieron TPI fue de 9471 apo. En el grupo que recibió TPI se presentaron 23 casos de TB (7,6% de 304) contra 305 en el grupo sin TPI (12,9% de 2374; *P* = 0,008). La eficacia del TPI fue 85% (IC95% de 76 a 91%).

**CONCLUSIÓN:**

Un seguimiento prolongado confirmó que la vacuna MVA85A no es eficaz a largo plazo en los lactantes, pero atribuyó al TPI una disminución de seis veces del riesgo de padecer TB. Los programas nacionales contra la TB de los países con alta carga de morbilidad deben velar por la aplicación óptima del TPI en los niños que cumplen las condiciones para recibirlo.

APPROXIMATELY ONE MILLION tuberculosis (TB) cases occurred globally in children in 2015.^1^ Isoniazid preventive therapy (IPT) is a key intervention for TB disease prevention. A meta-analysis showed IPT efficacy of 48% in non-human immunodeficiency virus (HIV) infected children.^2^ Despite recommendations that children aged <5 years with latent tuberculous infection (LTBI) or close contact with a TB patient should receive IPT, linkage to care is poor in high TB burden countries.^1^,^3^ Three South African studies showed that respectively only 20%, 28% and 33% of children referred for IPT actually received it,^4–6^ which is similar to an estimate from Ethiopia (33%).^7^ Health systems strengthening is needed to ensure IPT is administered when indicated.

Effective vaccination is another long-term strategy for TB control. An infant modified vaccinia Ankara virus-expressing antigen 85A (MVA85A) vaccine trial showed no protective benefit against TB disease over a median 2 years of follow-up.^8^ In the absence of short-term benefit, all vaccinees should ideally be followed for a longer period, not only to detect possible long-term protection, but also to identify any subsequent increased risk for TB disease.

Our primary objective was to describe the long-term effectiveness of infant MVA85A boost vaccination against TB. Secondary objectives were 1) to describe the impact of IPT on TB disease case accrual, and 2) to explore the durability of IPT protection.

## STUDY POPULATION AND METHODS

We analysed data from a double-blind, randomised, placebo-controlled phase 2b MVA85A infant TB vaccine trial (2009–2012) in HIV-negative South African children.^8^ We also obtained post-trial data from a regional electronic TB register (ETR) (2012–2014). Briefly, 4–6-month-old, bacille Calmette-Guérin (BCG) vaccinated, healthy infants were recruited, but excluded and referred in writing to public clinics for IPT, which is provided free of charge, if they had LTBI, TB disease or household contact with TB disease. Study staff were not empowered to enforce clinic attendance, IPT prescription or adherence to IPT. IPT was not a trial intervention but was provided as part of routine care. Public clinics are staffed by nurses and use standardised IPT and TB treatment algorithms. International and South African national guidelines do not recommend QuantiFERON^®^-TB GOLD In-Tube (QFT; Qiagen, Hilden, Germany) screening of young children as an indication for IPT in high TB burden countries. IPT consisted of 6 months of 10–15 mg/kg isoniazid (INH) taken five times weekly. Infants were randomised to receive MVA85A vaccine/placebo and followed for a median of 5 years for incident TB disease using a hierarchy of three endpoint definitions.^8^ This analysis uses the clinical endpoint definition—treatment for TB disease—to standardise the outcome of interest during and after the trial.

LTBI was defined as being positive on QFT or the tuberculin skin test (TST; positive if diameter ⩾ 10 mm) performed at enrolment, at day 336 and at the end of the trial for all children and at investigation for suspected TB disease. ‘Date of LTBI’ was defined as the earliest date of a positive test. Children who were TST/QFT-negative without subsequent conversion were classified as non-LTBI.

Two independent categories of exposure were defined, ‘vaccination’ (vaccine/placebo) and ‘IPT’ (documented to have started/initiated vs. not started), stratified by LTBI. Survival curves and incidence rates were calculated using survival analysis, whereas incidence rate ratios (IRRs) were calculated using Poisson regression.^9^ Survival time was calculated from the date of IPT initiation in children documented to have started IPT, from the date of LTBI in children with LTBI not documented to have started IPT, and from the date of the first negative TST/QFT result in non-LTBI children. For both vaccine and IPT effectiveness analysis, survival time ended on the date of the first diagnosis of TB disease, administrative censorship, death or migration, whichever was earliest.

Parents or legal guardians provided written informed consent for children to participate in the trial. As we obtained separate scientific and ethical approval from the University of Cape Town, Cape Town, South Africa, for this follow-up study, the need to re-contact all parents/guardians for new consent was waived.

## RESULTS

**Figure 1.**
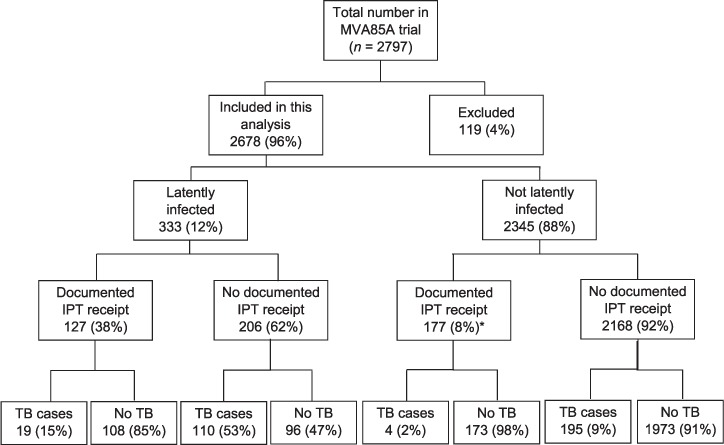
Flowchart of 2797 study participants. A total of 119 (4.3%) were excluded from the analysis of effectiveness of IPT, 40 (33.6%) of whom received IPT (20 had a missing date of IPT initiation and 20 developed TB disease within 2 months of IPT initiation; the latter were excluded because TB disease was presumed to be incipient at the time IPT was initiated and thus not attributable to IPT failure). Of 119 participants who did not receive IPT, 79 (66.4%) had no LTBI result (14 because one of three components of the QFT test result was missing, 40 had a missing date of testing for LTBI and thus survival time could not be calculated, and 25 had a survival time of 0 months; Poisson regression requires that data points with a survival time of 0 be excluded before analysis). Of all participants excluded, 106 had TB disease, 40 received IPT, 66 did not receive IPT, whereas 13 participants neither received IPT nor had TB disease. Thus, more excluded participants who received IPT had TB disease than those who did not receive IPT. * IPT started because of a history of household contact with a TB patient. IPT = isoniazid preventive therapy; TB = tuberculosis; QFT = QuantiFERON^®^-TB GOLD In-Tube; LTBI = latent tuberculous infection.

Of 2797 enrolled children, 1399 (50%) received the vaccine and 1398 (50%) the placebo. As previously reported,^8^ baseline demographic and clinical characteristics were comparable across the two groups, and 199 children discontinued follow-up early.^8^ Only 2678 children were included in the IPT analysis (Figure 1): 2138 (80%) were of mixed race, 534 (20%) were Black African, 3 (<1%) were Asian and 3 (<1%) were White. The median age at the end of the extended follow-up period was 4.8 years (interquartile range 4.4–5.2) and was comparable across vaccine/placebo arms. The median period between specimen collection for LTBI testing and IPT initiation was 49 days.

Of 2678 children, 333 (12.4%) had LTBI: 44 were positive on TST only, 146 on QFT only, and 143 on both TST and QFT. In total, 304 (11%) children were documented to have started IPT based on LTBI (*n* = 127, 38.1%) or close/household TB contact (*n* =177, 7.5%; Figure 1). IPT uptake was comparable across vaccine/placebo arms (*P* = 0.90). IPT was administered during the trial in 248 (81.6%) children, and post-trial in 56 (18.4%) children.

A total of 328 cases of TB disease were diagnosed: 19 (15.0%) among the 127 LTBI IPT recipients, 110 (53.4%) among the 206 LTBI non-IPT recipients, 4 (2.3%) among 177 non-LTBI IPT recipients, and 195 (9.0%) among 2168 non-LTBI non-IPT recipients. Of the 328 TB cases, 201 (61.3%) occurred by the age of 2 years. The overall incidence rate of TB disease was 3.2 cases/100 person-years of observation (pyo) (95% confidence interval [CI] 2.8–3.5).

### Risk of tuberculosis disease by MVA85A vaccine/placebo arm

**Figure 2.**
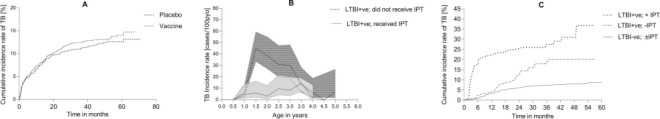
Effectiveness of MVA85A vaccine and IPT. A) Risk of TB disease by vaccine or placebo arm. ‘Time’ refers to the period since vaccination with MVA85A vaccine or placebo. B) Risk of TB disease by age and history of IPT among children with Mycobacterium tuberculosis infection. C) Cumulative risk of TB disease, stratified by M. tuberculosis infection and IPT. Cases of TB disease occurring within 2 months of documented initiation date of IPT are not included. Cumulative incidence rate of TB disease is presented as the number of cases/100 pyo. Incidence rates were estimated based on the number of TB disease cases/100 pyo. Rates were calculated using 6-monthly intervals from birth. Shaded areas represent 95% confidence intervals for incidence rate estimates. Children were recruited at 4–6 months of age, hence the low rate of TB disease in children aged <1 year. TB=tuberculosis; IPT=isoniazid preventive therapy; MVA85A=modified vaccinia Ankara virus-expressing antigen 85A; LTBI=latent tuberculous infection; pyo=person-years of observation.

Incidence of TB disease was 3.3/100 pyo (95%CI 2.9–3.9) in the MVA85A vaccine arm and 3.0/100 pyo (95%CI 2.6–3.5) in the placebo arm (*P* =0.397) (Figure 2). The hazard ratio for TB disease for vaccine vs. placebo arm was 0.96 (95%CI 0.68–1.36) and vaccine efficacy was 4% (95%CI −36 to 32).

### Risk of tuberculosis disease among children with LTBI stratified by IPT uptake

Analysed by 6-monthly age bands and stratified by IPT administration and LTBI status, TB disease incidence was highest among children aged 1.0–2.5 years who did not receive IPT (Figure 2). Thereafter, TB disease incidence among IPT recipients approximated that among non-IPT recipients. The incidence rate of TB disease among children with LTBI who did not receive IPT (33 cases/100 pyo) was more than six times that of children with LTBI who received IPT (5 cases/100 pyo) (Table 1).

**Table 1:**
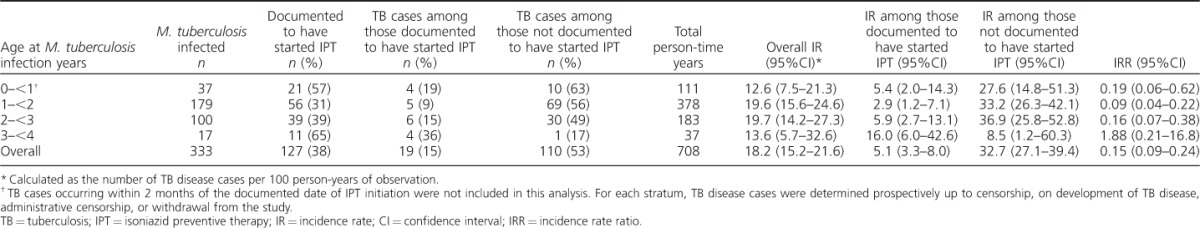
Risk of TB disease stratified by age at first diagnosis of Mycobacterium tuberculosis infection

Compared with non-LTBI children, IPT administration reduced the risk of TB disease from 16-fold (IRR 15.82, 95%CI 12.54–19.97) among LTBI children who did not receive IPT to 2.5-fold (IRR 2.48, 95%CI 1.55–3.97) in LTBI children who received IPT, after adjusting for parent-reported racial ancestry. The risk of TB disease among mixed-race children was more than two-fold (IRR 2.17, 95%CI 1.54–3.07) that of children with Black African ancestry, after adjusting for IPT uptake.

Among children with LTBI, we found no confounding effect on the relationship between TB disease and IPT by vaccination status (MVA85A vaccine or placebo), sex, age at enrolment or age at LTBI (Table 2). In LTBI children, there was no statistically significant effect modification between IPT uptake and age at LTBI, after adjusting for racial ancestry (data not shown). The model of best fit selected by stepwise comparison of Akaike's Information Criterion and the likelihood ratio test statistic showed that among children with LTBI, IPT reduced the risk of TB disease by 85% (95%CI 76–91, *P* < 0.001) after adjusting for racial ancestry, whereas the risk of TB disease was 65% lower among children with Black African ancestry than among mixed-race children (95%CI 34–81, *P* = 0.001), after adjusting for IPT uptake.

**Table 2:**
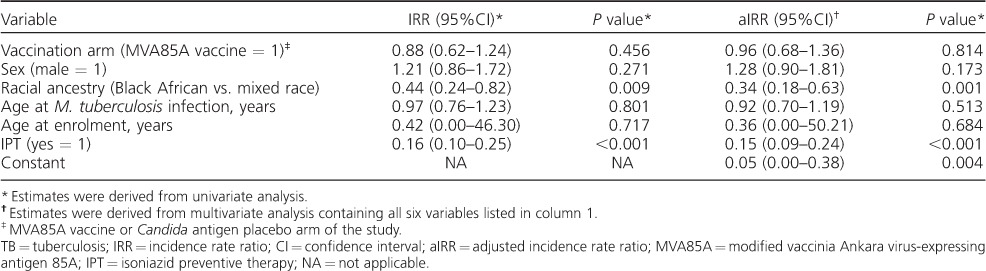
Univariate and multivariate analysis of factors associated with the risk of TB disease

### Exploration for MVA85A vaccine/isoniazid preventive therapy effect modification

Among MVA85A vaccine recipients, IPT reduced the risk of TB disease by 85% (95%CI 76–97) whereas, in placebo recipients, IPT reduced the risk of TB disease by 64% (95%CI 39–79, *P* = 0.032).

### Durability of protection afforded by isoniazid preventive therapy

Among LTBI children, the protective benefit of IPT was transient. IPT afforded protection against TB disease during and approximately 6 months after completion of IPT; however, this protective benefit decayed rapidly over the 6 months to 1 year following the expected date of IPT completion (Figure 2). We performed an exploratory analysis to examine a possible rebound in risk of TB disease occurring 1.5–2.5 years after the documented start of IPT in LTBI children. Compared with LTBI children who did not receive IPT, there was no statistically significant difference in risk over this period (data not shown).

## DISCUSSION

Our findings confirm that infant MVA85A boost vaccination neither provides added protection to BCG against TB disease nor does it adversely affect TB disease risk up to 5 years after vaccination.^8^ This study population had an exceedingly high incidence of TB disease, which was greatest in children with LTBI who bore more than one third of the disease burden. Although IPT was 85% protective, IPT did not completely reduce the TB risk in LTBI children to that of non-infected children; children with LTBI who received IPT were still 2.5 times more likely to develop TB disease than non-LTBI children. Furthermore, the protective effect of IPT waned rapidly beyond 6 months after the expected date of completion of IPT. Children who became infected, but who did not receive IPT, were at highest risk of TB disease between the ages of 1 and 2.5 years, after which time TB disease risk approximated that of IPT recipients. It is also notable that racial ancestry played an important part in susceptibility to childhood TB in this region, with children of mixed-race ancestry having more than twice the risk of TB disease by the age of 5 years than Black African children, even after adjusting for IPT. This finding is supported by our previous data from a large cohort study of adolescents in the same study community.^10^

The high cumulative risk of TB disease among children with LTBI who were not documented to have started IPT (33%) is similar to that reported by Trauer et al.^11^ (36%) and Sloot et al.^12^ (33%). Pre-chemotherapy-era studies also showed that 10–30% of young children with LTBI develop disease.^13^ The impact of IPT, which has clear protection against TB disease and is thus ethically mandatory for LTBI children, should therefore be considered carefully when estimating TB case accrual and sample size in future TB efficacy vaccine trials. Although we showed a modest difference in the effectiveness of IPT by study arm, with IPT being approximately 20% less effective in the placebo arm, we are unable to explain this observation, which may be a chance finding. We estimate that IPT prevented 62 TB cases among LTBI children in this trial, whereas an additional 127 TB cases could potentially have been prevented if there had been 100% IPT uptake. However, given the modest uptake of IPT in this study despite written referral and adherence support, health systems in developing countries may struggle to achieve optimal coverage. It must also be acknowledged that current international and South African national guidelines do not recommend the use of QFT screening as an indication for IPT in young children living in TB-endemic countries. High rates of TB transmission in high TB burden countries also lead to the risk of reinfection starting immediately following completion of IPT, resulting in a limited impact of IPT after completion of treatment.^14–17^

We previously reported a lower effectiveness of IPT (52%) among a smaller group of infants with a history of household TB exposure and/or LTBI.^4^ Differences in these estimates might be explained by differences in study population and the denominator and a clinical trial effect, whereby participants in this study were more rigorously followed and investigated for TB disease. However, the 85% effectiveness of IPT among children with LTBI reported here is equivalent to the 88% reported by Trauer et al.^11^

The message for national health systems and TB control programmes in high-burden developing countries is clear: IPT administration to children with LTBI reduces the risk of TB disease from 16-fold to 2.5-fold that of non-infected children, with one case of TB disease prevented for every four children with LTBI who were documented to have started IPT. This important finding should act as a powerful stimulus for more rigorous and effective implementation of TB contact tracing and IPT policies to prevent childhood TB in high-burden countries.

Our finding that MVA85A boost vaccination did not alter TB disease risk up to 5 years of age is based on the third, least rigorous clinical trial case definition: provision of anti-tuberculosis treatment by an attending clinician.^8^ This was a pragmatic approach, as post-trial follow-up relied on health service data and we did not have access to standardised clinical, radiographic and microbiological investigations to apply the most rigorous case definitions. TB incidence in the post-trial period may therefore have been underestimated by the use of passive surveillance compared with the in-trial incidence obtained from active surveillance. However, we do not anticipate that this approach would have biased estimation of the vaccine or IPT effects, which would have been affected equally. It is also possible that the clinical TB case definition included false-positives in estimates of TB disease rates and tend to negate true differences in incidence between groups. It is therefore notable that the clinical TB disease endpoint was sufficiently robust to detect statistically significant and clinically meaningful differences in TB disease incidence in IPT recipients and non-recipients, and in children from different parent-reported racial ancestries.

The higher risk of TB disease among mixed-race children than in Black African children persisted after adjusting for confounders. A small number of studies have reported a genetic predisposition to TB disease.^18–22^ This has been attributed to the *NRAMP1*, *VDR* and *MBL* genes, among others,^18^ although results have been inconsistent,^22^ possibly because non-genetic host factors, environmental factors and pathogen virulence factors obscure true genetic associations.

A limitation of our analysis is that LTBI was defined using TST and QFT results obtained during the follow-up period of the trial. It is certain that additional LTBI occurred in the post-trial period and may have been associated with observed TB disease cases. This factor might have resulted in overestimation of TB incidence rates in non-LTBI children and underestimation of rates in LTBI children, but is unlikely to have impacted the association between racial ancestry and TB disease, or impact of IPT. We believe that the transient beneficial effect of IPT is explained by the high level of infection in our setting, which may limit the generalisability of our findings to populations with much lower rates of TB transmission. The nature of data obtained from the ETR did not allow us to precisely define the follow-up time. Post-trial changes in key identifiers, such as names, might have resulted in missing case records and corresponding underestimation of TB disease incidence. IPT was administered in non-trial conditions and adherence data were not available, nor were data on potential confounders other than sex, age and race; we therefore adjusted only for these available potential confounders. We also assumed that all children who did not develop TB disease survived until the date of censorship.

Despite these limitations, our data send a powerful message to public health officials in TB-endemic, resource-limited countries. IPT has the potential for a six-fold reduction in TB disease rates among young children with LTBI and should be implemented optimally and without further delay. The message for childhood TB research programmes is equally clear. IPT is integral to the standard of care for children in TB-endemic countries and the impact of IPT must be carefully considered in the design of TB vaccine clinical trials.
